# Home is where the ice is: polar bears teach us about the Arctic

**DOI:** 10.1093/conphys/coab013

**Published:** 2021-03-16

**Authors:** Madeleine S Killacky

**Affiliations:** Bangor University, Bangor, Gwynedd, LL57 2DG, UK

Polar bears depend on the Arctic sea ice for survival. In the cold, frozen tundra, deep snow and ice, they hunt for food, find mates and teach their young survival skills. But, it turns out that polar bears can also teach us humans a lot about the long-term health of the Arctic environment.

Every year, polar bears (*Ursus maritimus*) cycle through predictive stages of living on ice and on land within their native range, which spans the Arctic Circle. While most polar bears are born on land, they depend on sea ice for feeding—they mostly eat seals—and therefore survival, and so polar bears are classed as marine animals. Polar bears also sit at the top of the food chain and as hypercarnivores, more than 70% of their diet consists of meat. For scientists, such as Amy Johnson and her colleagues, these traits make polar bears even more interesting as indicator species. Changes at the top of a food chain often suggest that there are more serious changes happening at lower levels in the food chain. Indeed, these are also some of the reasons why polar bears are classified as ‘vulnerable’, because if they are not protected, the entire Arctic ecosystem faces collapse, which is entirely possible. Since the last decade of the 20th century and over the first two decades of the 21st century, the Arctic has experienced rapid and extensive changes to its sea ice levels.

To make some of these connections between polar bear health and ecosystem health, Johnson and her team investigated the Western Hudson Bay polar bear population, where a long-term monitoring program had been capturing, marking and measuring the animals over three decades (1985–2018). The team’s objectives were to assess the polar bears’ energy levels, which were estimated mathematically using body measurements, over time to determine the influence of changing sea ice levels. This trend could highlight how a changing environment can have lasting impacts on polar bear health. The team also investigated whether vulnerability to declining sea ice levels was influenced by age or sex of the polar bears.

The team’s analyses concluded that the polar bears’ energy declined by 53% over the 33-year period. Moreover, between 1987 and 2011, the total population declined by about 32%, from approximately 1185 to 806 polar bears. From these data, Johnson and her team found that solitary adult females were more affected by the changing environment than adult male bears. The data also showed that sea ice was breaking up for longer periods each year, which means that these bears have less access to their natural, ice habitat. If this trend continues, then polar bears risk reduced survival rates for their young and decreased reproductive success.

Johnson and her colleagues were able to link declining physiological health of the polar bears and the loss of ice in the Arctic. Such changes are likely already affecting the health of the entire Arctic ecosystem. It is, therefore, extremely important that scientists like Johnson and her colleagues continue to monitor the health of individual animals and their wider populations so that they can better understand the current health of whole ecosystems like the Arctic.

Illustrations: Erin Walsh, ewalsh.sci@gmail.com


